# From science to society: Exploring the nexus between obesity research and public awareness in Brazil

**DOI:** 10.1016/j.heliyon.2024.e37968

**Published:** 2024-09-18

**Authors:** Sofia Lafetá Pinto Santos, Renata Miyabara, Rym Ghimouz, Mirela Dobre, Andrei Brateanu, Luciana Aparecida Campos, Ovidiu Constantin Baltatu

**Affiliations:** a UNINOVAFAPI University Center, Teresina, Piauí, Brazil; b Center of Innovation, Technology, and Education (CITE) at Anhembi Morumbi University, Anima Institute, Sao Jose dos Campos Technology Park, São José dos Campos, Brazil; c College of Natural and Health Sciences, Zayed University, Abu Dhabi, United Arab Emirates; d Division of Nephrology and Hypertension, University Hospitals, Cleveland, OH, United States; e Medicine Institute, Cleveland Clinic, Cleveland, OH, United States; f College of Medicine, Alfaisal University, Riyadh, Saudi Arabia

**Keywords:** Obesity, Brazil, Public awareness, Scientific publications, Web search trends

## Abstract

Raising public awareness about the medical aspects of obesity is crucial in Brazil, given its escalating prevalence. This study investigated the correlation between scientific research on obesity in the country and public awareness by scrutinizing scientific publications and online search trends. Scientific data were sourced from Dimensions, while online engagement data were extracted from Google Trends. Key metrics were analyzed, including scientific publication trends, Altmetrics, news image trends, YouTube trends, and web search trends. Linear regression and the Mann-Kendall test assessed trends, and Principal Component Analysis (PCA) explored dataset variations.

Scientific publications on obesity in Brazil consistently increased from 2004 to 2023, reflecting growing scientific interest. The number of publications increased from 300 in 2004 to 7566 in 2022, representing a 25-fold increase. However, web search trends among the general population declined by 79 % during the same period, indicating ineffective "knowledge translation”. Altmetrics and news image trends fluctuated, while YouTube trends exhibited a decline. The disparity between scientific publications and public web search trends highlights the necessity for more efficient scientific information communication.

The gap between scientific publishing and public web search trends highlights the need for improved scientific communication. Measures such as formal science communication training for scientists, leveraging social media, embracing arts-based approaches, and nurse advocacy can facilitate informed public discourse and foster interest in obesity-related topics.

## 1 Introduction

Obesity is a global health crisis affecting millions of people worldwide. According to a recent systematic Global Burden of Disease Study analysis, the age-standardized global DALY rates attributable to high body-mass index (BMI) rose by 15.7 % (9.9–21.7) between 2000 and 2021 [1]. This epidemic affects not only high-income countries; low- and middle-income countries also have to deal with a "double burden of malnutrition" (DBM), which is the simultaneous presence of undernutrition and overnutrition [2]. Latin America has experienced a faster shift from a mainly underweight to an overweight and obese population than other areas in the world [2]. The global nature of this issue underscores the importance of understanding obesity trends and public awareness in diverse contexts, including emerging economies like Brazil.

Public awareness of obesity in Brazil is an essential health and public policy issue. The country is in the early stages of addressing obesity, and more efforts are needed to curb the progression of the disease [3]. We and other researchers have identified a growing prevalence of childhood obesity and metabolic syndrome in Brazil [4,5]. In recent decades, higher rates were observed in boys, especially in the more developed regions [5]. Among Brazilian adults, the prevalence of obesity has also been on the rise, particularly among women with secondary education [6]. From 2002 to 2013, the prevalence of obesity in Brazil increased from 7.5 % to 17.0 % among adults aged 20–39 years and from 14.7 % to 25.7 % among those aged 40–59 years [6]. If the current trends of body mass index (BMI) increase are maintained from 2021 to 2030, approximately 5.26 million incident cases and 808.6 thousand deaths from non-communicable diseases (NCDs) may occur due to overweight [7].

Contributors to the high rate of obesity in Brazil include contextual and socio-economic factors, lifestyle choices, and demographic characteristics such as demographic density, employment rates, Gross Domestic Product (GDP) per capita, income inequality (Gini coefficient), urbanization rate, level of education, and income status [8]. Additionally, age, living with a partner, education level, income, and smoking status are associated with obesity among industrial workers in Brazil [9]. Socioeconomic inequality, income, demographics, schooling, and lifestyle factors also contribute to the prevalence of overweight and obesity in the Brazilian adult population [10]. In rural areas, factors such as socioeconomic class, travel time to purchase food, age, dietary patterns, and workload are associated with obesity [11]. Furthermore, the number of McDonald's restaurants has a strong positive correlation with overweight and obesity rates, particularly among children and adolescents in Brazil [12].

Obesity in Brazil has significant social and economic consequences. At the individual level, obesity is associated with reduced life expectancy, lower productivity and wages, increased healthcare expenditure, and private expenditures on preventative measures [10]. The economic consequences of obesity extend beyond medical costs and include indirect or social costs such as decreased quality of life, social adjustment problems, productivity loss, premature retirement, and death [9]. Additionally, socioeconomic factors play a role in obesity, with lower education levels, lower income, and physical inactivity being associated with higher obesity rates [13]. In terms of socioeconomic inequality, the concentration of overweight and obesity is higher among the richest men and rich women in less developed regions, while poor women in more developed regions are also affected [14]. These findings highlight the need for public policy strategies that address the persistence of obesity, promote equity and equality in health, and consider the sociodemographic characteristics of the population.

Factors contributing to the public awareness of obesity in Brazil include the shifts in dietary consumption and energy expenditure influenced by economic, demographic, and epidemiological changes at a population level [3]. Additionally, the lack of attention to the healthcare of children and adolescents in many parts of the world highlights the importance of research and activities focused on improving healthcare education and fostering healthy habits [15]. Socioeconomic inequality, including income, demographics, schooling, and lifestyles, also plays a role in the prevalence of overweight and obesity in the Brazilian adult population [10]. The implementation of comprehensive national inter-sectorial plans, food and nutrition education frameworks, and promotion of healthy food and physical activity in various settings are important initiatives to address the rising rates of overweight and obesity in Brazil [16].

Strategies for dealing with obesity in Brazil involve both the healthcare system and the food and nutritional security system, emphasizing the need for integrated and intersectoral approaches [17]. The Brazilian Unified National Health System (SUS) approaches obesity as both a risk factor and a disease, focusing on changing eating practices and physical activity. The Food and Nutritional Security System (SISAN) views obesity as a social problem related to food insecurity and proposes integrated approaches to change eating practices.

New technologies and data sources in the digital age offer unique possibilities to study social trends and public interest in health issues. Platforms like Google Trends provide real-time data on search behaviors, allowing researchers to monitor and examine public interest in ways that traditional survey methods cannot. For example, Bayram and Ozturkcan used Google Trends data from 2004 to 2022 to identify public interest trends in weight loss, physical activity, diet, nutrition, healthy diet, healthy nutrition, optimum nutrition, healthy food, and a combination of weight loss and diet-related topics in Europe. Comprehensive research information databases like Dimensions [18,19] can help gain a more nuanced understanding of the scientific research output of critical health issues like obesity in Brazil. Using these technologies in health research is essential for detecting trends, informing public health strategies, and narrowing the gap between scientific knowledge and public awareness.

This study aimed to investigate the relationship between the scientific interest in obesity and the public discussions and level of awareness of this condition. Thus, we aimed to understand the interplay between scientific knowledge dissemination and public awareness in the context of obesity in Brazil.

## 2 Methods

### 2.1 Research framework and design

To investigate the relationship between scientific research on obesity in Brazil, public awareness, and online engagement, we employed the SPIDER framework [20]:

• *Sample*: The scientific publications on obesity in Brazil from 2004 to the present (retrospective observational aspect) and the discussions related to obesity in Brazil on social media platforms during the same time period (data science aspect).
• *Phenomena of Interest:* The relationship between scientific research on obesity in Brazil and the levels of public awareness and discussions regarding obesity on social media platforms.
• *Design*: A hybrid approach, combining retrospective observational analysis of scientific publications and data science techniques for online engagement data
• *Evaluation*: Assessing the correlation between the trends in scientific research output on obesity in Brazil and the corresponding levels of public awareness and discussions on social media. Statistical analyses conducted for both scientific publications and online engagement data.
• *Research Type*: Multidisciplinary investigation, blending elements of retrospective observational research with data science techniques.

### 2.2 Data collection and sources

We conducted a comprehensive data collection process utilizing two distinct sources:

**Scientific Data:** We accessed Dimensions, a comprehensive multidisciplinary research platform housing scholarly publications, grants, clinical trials, and Altmetric attention scores [21,22]. To collect metrics from scientific literature in both Portuguese and English, we conducted searches using Boolean terms "obesidade AND Brasil" OR "obesity AND Brazil," restricting the search to research organizations located in Brazil and the time period from 2004 to the present. The data was obtained on Sep 30, 2023, from Digital Science's Dimensions platform, available at https://app.dimensions.ai. Access was granted to subscription-only data sources under a license agreement.

**Online Engagement Data:** To gain a comprehensive overview of obesity's popularity in Brazil over time, we utilized Google Trends. Our search focused on the term "Obesidade" within the "Web" category and the location "Brazil." The "Web" category encompasses data from various websites, including news websites, blogs, forums, and social media platforms.

### 2.3 Key metrics

Primary key metrics included:

• Scientific publication trends and
• Web search trends among the public.

### 2.4 Secondary measures included

• Scientific metrics:
  o Citations: Total publication citations are the number of times that other publications in the database have cited a publication.
  o Median number of citations per publication published in each year.
  o Grants: number of grants active and the number of grants started in each year.
  o Clinical trials: number of clinical trials active and the number of clinical trials started in each year
• Public interest metrics:
  o Publications with attention: the percentage of publications with Altmetric attention.
  o Altmetric Attention Score: The mean Altmetric Attention Score is a weighted count of all the online “attention” Altmetric has discovered for an individual research output. This includes mentions in public policy documents as well as references in Wikipedia, mainstream news, social networks, blogs, and other places.
  o Web news search (analytics only available starting 2008): Tracked the search popularity of the term "obesidade" in the Brazilian news articles over time to see which obesity stories are resonating with the public.
  o Web image search (analytics only available starting 2008): Tracked the search popularity of images related to obesity over time to see how people in Brazil are visually engaging with the topic of obesity.
  o YouTube search (analytics only available starting 2008): Tracked the search popularity in Brazil of videos about obesity over time.

To explore potential correlations between trends in research output metrics related to obesity in Brazil and corresponding metrics of online engagement, we conducted the following statistical analyses:

• Linear regression analysis - to quantify trends in the popularity metrics over time. For each metric, a linear trend line was fitted to the data, and the slope of this trend line was calculated. The significance of each slope was assessed to determine whether it differed significantly from zero, indicating a non-zero trend. DFn (Degrees of Freedom numerator) and DFd (Degrees of Freedom denominator) were used to represent degrees of freedom, which are critical for calculating F-statistic and p-value and determining critical values for hypothesis testing.
• Mann-Kendall test – to detect monotonic trends in time series data. It assessed the presence of trends and determined their direction, whether positive (increasing), negative (decreasing), or stable (no significant trend).
• *Principal Component Analysis (PCA)* - to explore significant variations within the dataset. By examining the magnitude of eigenvalues and eigenvectors, PCA identified the principal components that contributed most to the variance in the data. Statistical significance was determined at a 5 % level (p < 0.05), indicating that any correlations observed would be deemed statistically significant if the probability of a Type I error was less than 5 %. All statistical analyses were executed using GraphPad Prism version 8.1.2 for Mac OS X (GraphPad Software, La Jolla California USA) and MatLab MATLAB. (2022a). Natick, Massachusetts: The MathWorks Inc.

Before analyses, we assessed the normality of the data distribution using the D'Agostino & Pearson test.

## 3 Results

### 3.1 Scientific versus public interest trends

**Fig. 1:**
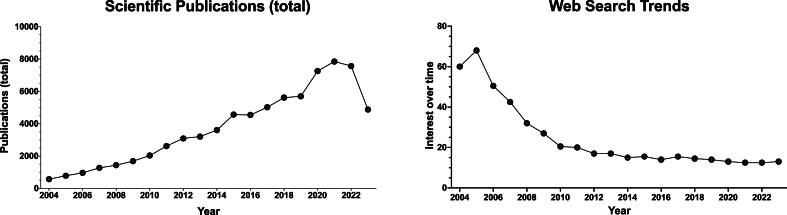
**Bibliometric data on Scientific publications (total) trends and web search trends.** Left panel: number of publications published in each year; Right panel: Interest over time on Web search: The numbers represent search interest in comparison to the chart's maximum point for the given region and time. A value of 100 represents the term's peak level of popularity. The 2023 data does not represent a full year and should not be interpreted as a decline in publication volume.

Analysis of scientific publication trends from 2004 to 2023 revealed a notable and consistent increase in the number of research articles related to obesity in Brazil (Fig. 1). Despite the 2023 data representing only a partial year (January to September), its inclusion in the analysis did not negatively impact the significance of our results. In contrast to this growing scientific interest, there has been a striking decline in the general public's interest in obesity-related web searches over the same period (2004–2023).

In the simple linear regression analysis of scientific publications (total), a significant positive linear relationship was observed, with a slope of 413.3 (95 % Confidence Interval (CI): 344.2 to 482.3, p < 0.0001). Conversely, the Web Search Trends data revealed a significant negative linear relationship, with a slope of −3.340 (95 % CI: 4.654 to −2.027, p < 0.0001). These findings indicate that "Publications (total)" and "Web Search Trends" are moving in opposite directions and differ in terms of linearity.

In Principal Component Analysis (PCA), the first principal component (PC1) was significantly influenced by both "Publications (total)" and "Web Search Trends." The eigenvector for "Publications (total)" was 0.7071, indicating a strong positive contribution to PC1. This suggests that as the number of total publications increases, so does the value of PC1. Conversely, "Web Search Trends" had an eigenvector of −0.7071, demonstrating a strong negative contribution to PC1. This implies an inverse relationship, where an increase in web search trends corresponds to a decrease in PC1. The equal but opposite eigenvectors for these two variables highlight a notable negative correlation within the context of PC1.

### 3.2 Scientific data trends over the last two decades

**Fig. 2:**
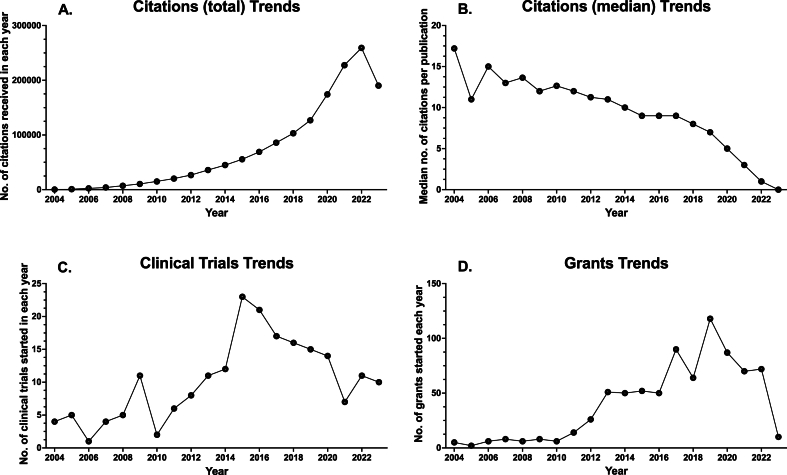
**Bibliometric data on scientific data trends. A.** Total number of citations received in each year (the total number of times that publications have been cited by other publications in the database); **B.** Median number of citations per publication published in each year (the median values of the number of times that publications have been cited by other publications in the database); **C.** Starting clinical trials (the number of grants started in each year); **D.** Starting grants (the number of grants started in each year). The 2023 data does not represent a full year and should not be interpreted as a decline in scientific data metrics.

The search of the Dimensions research information database yielded 74,304 papers, 203 clinical trials, and 795 grants (Supplement 1, Excel Book “Obesity in Brazil”, sheets “Dim. Publications”, “Dim. Clinical Trials”, “Dim. Patents”, and “Dim. Grants”). There was a concurrent increase in the yearly number of citations with the growing number of papers linked to obesity in Brazil, indicating a persistent scholarly interest in the topic (Fig. 2A). However, the median number of citations per publication exhibited a declining trend from 2004 to 2024 (Fig. 2B). Notably, there was substantial growth in the number of active clinical trials and grants pertaining to obesity research in Brazil, particularly in the second decade of the investigation (Fig. 2C and 2D).

### 3.3 Top-published research organizations

The leading research organizations in terms of obesity-related publications include Universidade de São Paulo (USP) with 15,954 publications to date, Federal University of São Paulo (UNIFESP) with 6045 publications, and State University of Campinas (UNICAMP) with 5468 publications (Supplement 1, Excel Book "Obesity in Brazil," sheet "Dim. Publications ResOrg").

### 3.4 Top funders for obesity publications

The primary funders contributing to obesity-related publications are the National Council for Scientific and Technological Development (CNPq) with 16,891 publications to date, the Coordenação de Aperfeicoamento de Pessoal de Nível Superior (CAPES) with 13,866 publications, and the São Paulo Research Foundation (FAPESP) with 8659 publications (Supplement 1, Excel Book "Obesity in Brazil," sheet "Dim. Publications Funders").

### 3.5 Top funders for grants

In terms of funding grants related to obesity research, the most prominent organizations are the Coordenação de Aperfeicoamento de Pessoal de Nível Superior (CAPES) with 427 funded grants to date, the National Council for Scientific and Technological Development (CNPq) with 182 funded grants, and the São Paulo Research Foundation (FAPESP) with 177 funded grants (Supplement 1, Excel Book "Obesity in Brazil," sheet "Dim. Grants Funders").

### 3.6 Online engagement data trends over the last two decades

**Fig. 3:**
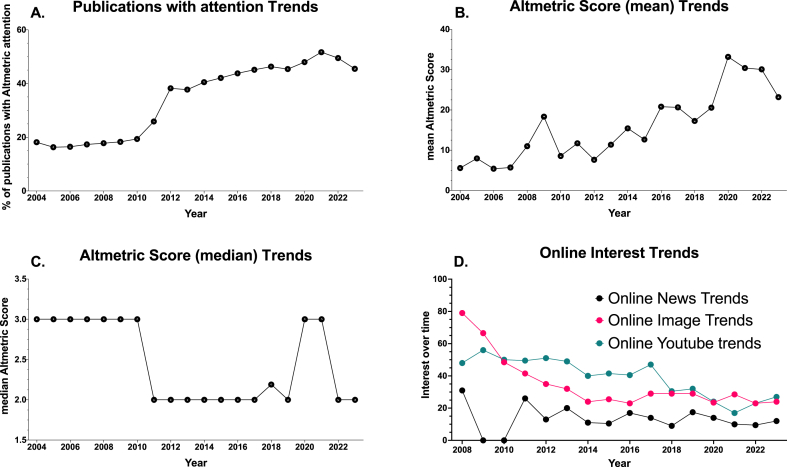
**Bibliometric data on public interest. A.** Publications with attention: percentage of publications with Altmetric attention published in each year; **B.** Mean Altmetric Attention Score for all publications with attention published in each year; **C.** Median Altmetric Attention Score for all publications with attention published in each year; **D.** Online News Trends; **E.** Online Image Trends; **F.** Online YouTube Trends. The Altmetric Attention Score is a weighted count of all of the online attention Altmetric has found for an individual research output. This includes mentions in public policy documents and references in Wikipedia, mainstream news, social networks, blogs, and more. The 2023 data does not represent a full year and should be interpreted cautiously compared to previous years.

Analysis of key metrics demonstrated distinct trends in scientific and public interest in obesity in Brazil (Fig. 3A-D and Table 1). Scientific interest indicators, including Publications (total) (Fig. 1, Table 1), Citations (total) (Fig. 2A., Table 1), Grants (total) (Fig. 2D., Table 1), and Clinical trials (total) (Fig. 2C., Table 1), exhibited an upward trend, indicating a growing scientific emphasis on obesity research. Conversely, metrics representing the general Brazilian public's interest in obesity, such as Publications with attention (%) (Fig. 3A.) and Altmetric Attention Score (mean) (Fig. 3B.), also showed an increasing trend, signifying rising public engagement with scientific research on obesity.

**Table 1:** Mann-Kendall trend, linear regression slope analysis, and eigenvectors of variables on the First Principal Component (PC1). Coef.Var. = coefficient of variation; Std. Error Slope = Standard Error Slope; DFn = Degrees of Freedom numerator; DFd = Degrees of Freedom denominator.

Key Metrics	Mann-Kendall				Linear Regression: Is slope significantly non-zero?					
	Trend	S	Coef.Var.	Confidence Factor	Slope	Std. Error Slope	F	DFn, DFd	P value	Deviation from zero?
Publications (total)	Increasing	174	0.62	>99.9 %	372,7	28.98	165.4	1, 18	<0.0001	Significant
Citations (total)	Increasing	186	1.11	>99.9 %	12463	1370	82.7	1, 18	<0.0001	Significant
Publications with attention (%)	Increasing	168	0.40	>99.9 %	2.14	0.18	135.7	1, 18	<0.0001	Significant
Altmetric Attention Score (mean)	Increasing	134	0.54	>99.9 %	1.27	0.17	55.5	1, 18	<0.0001	Significant
Grants (total)	Increasing	129	0.89	>99.9 %	4.40	0.95	21,4	1, 18	0,0002	Significant
Clinical trials (total)	Increasing	81	0.61	99.6 %	0.61	0.20	9.7	1, 18	0.0061	Significant
Online News Trends	Stable	−18	0.60	77.5 %	−0.25	0.45	0.3	1, 14	0.5888	Not Significant
Altmetric Attention Score (median)	Decreasing	−59	0.20	97.1 %	−0,05	0.02	7.5	1, 18	0.0136	Significant
Online Image Trends	Decreasing	−79	0.47	>99.9 %	−2.67	0.59	20.8	1, 14	0.0004	Significant
Citations (median)	Decreasing	−167	0.47	>99.9 %	−0.71	0.07	113.3	1, 18	<0.0001	Significant
Web Search Trends	Decreasing	−171	0.69	>99.9 %	−2.39	0.38	39.9	1, 18	<0.0001	Significant
Youtube trends	Decreasing	−86	0.31	>99.9 %	−2.27	0.29	61.1	1, 14	<0.0001	Significant

However, general public interest in obesity in Brazil, as represented by Online Image Trends, Web Search Trends, and YouTube trends (Table 1), showed a decreasing trend, except for Online News Trends (Fig. 3D.), which remained stable. This suggests a decline in the general public's interest in obesity. Notably, the Altmetric Attention Score (median) (Fig. 3C.) and Citations (median) (Fig. 2B., Table 1), representing the typical level of public interest in scientific research on obesity and the typical level of scientific citations, respectively, both showed a decreasing trend.

Table 1 provides a comprehensive analysis of trends and significance for key metrics related to obesity research in Brazil. The Mann-Kendall test reveals the direction of trends, indicating whether variables are increasing, stable, or decreasing. The linear regression analysis determines if the slope is significantly non-zero, indicating the strength and significance of the observed trend. Notably, total publications, citations, publications with attention, mean Altmetric Attention Score, grants, and clinical trials exhibit a consistent increasing trend, all statistically significant. Conversely, online news trends demonstrate stability and lack statistical significance. However, variables such as the median Altmetric Attention Score, online image trends, citations median, web search trends, and YouTube trends show a significant decreasing trend, emphasizing the need for effective strategies to reverse these trends and improve the translation of scientific knowledge into public awareness.

In the Principal Component Analysis (PCA), eigenvectors for the first principal component (PC1) were calculated (Table 2). These eigenvectors represent the directions in the new dimensional space where there is the most variance, indicating the variables contributing significantly to the variance of the data along PC1. Variables such as "Publications (total)," "Starting clinical trials (total)," "Starting grants (total)," "Citations (total)," and "Publications with attention (%)" had positive eigenvector values, indicating positive correlations with PC1. Conversely, variables such as "Citations (median)," "Web Search Trends," "Online Image Trends," "Online Youtube trends," and "Altmetric Attention Score (median)" had negative eigenvector values, indicating a negative correlation with PC1. This implies a strong relationship between scientific publications and publications with media attention, suggesting that publications receiving media attention are more likely to be associated with public interest.

**Table 2:** **Eigenvectors of Variables on the First Principal Component (PC1).** This table presents the eigenvectors, or loadings, of each variable on the first principal component (PC1) from a Principal Component Analysis (PCA). Each value represents the weight of the corresponding variable on PC1, which can be interpreted as the correlation between the original variables and the principal component. Positive values suggest that the variable tends to increase along with PC1, while negative values suggest that the variable tends to decrease as PC1 increases. The magnitude of these values indicates the strength of the correlation, with larger absolute values indicating stronger correlations.

Eigenvectors	PC1
Publications (total)	0.36
Publications with attention (%)	0.36
Citations (total)	0.33
Altmetric Attention Score (mean)	0.30
Starting grants (total)	0.28
Starting clinical trials (total)	0.17
Online News Trends	−0.06
Altmetric Attention Score (median)	−0.11
Online Image Trends	−0.31
Citations (median)	−0.33
Web Search Trends	−0.33
Youtube trends	−0.33

The analysis of obesity interest in Brazil using Google Trends showed a decline over the investigated period, with variations observed across geographic regions in Brazil. The figures defining interest over time represent search interest relative to the chart's peak for the given region and time (a value of 100 represents the term's peak level of popularity). The top states in Brazil where obesity was of interest were Piauí, Bahia, and Amapá (Supplement 1, Excel Book "Obesity in Brazil," sheet "Google Trends GeoMap Obesidade"). The geographic region where the term "obesidade" was most popular during the given time period was determined based on values calculated on a scale from 0 to 100, with 100 indicating the place with the highest popularity as a percentage of all searches in that location and 50 representing a place that is half as popular.

## 4 Discussion

The analysis of scientific publication trends from 2004 to 2023 revealed a noteworthy and consistent increase in the number of research articles related to obesity in Brazil. This upward trajectory reflects the growing attention of the scientific community to the issue of obesity within the country. The expansion of research in this domain signifies an intensified effort to understand, address, and mitigate the complexities associated with obesity. In contrast to the rising scientific interest, a striking decrease in web search trends related to obesity among the general public during the same time frame (2004–2023) was observed. This decrease in public web searches signifies a potential lack of efficient translation of scientific knowledge and findings to the broader population.

### 4.1 Increasing scientific publications, decreasing public interest

The results of this study indicate that there is a discrepancy between scientific publications and public interest on the topic of obesity in Brazil. While there has been an increasing trend in scientific publications on obesity in Brazil, there has been a decreasing trend in web searches among the public on this topic. This disconnect suggests that there is a lack of efficiently translating the science to the public. This is a concerning finding, as it means that the public may not be aware of the latest research on obesity and its prevention and treatment, underscoring the need for more effective science communication and outreach efforts. Bridging this gap is essential to ensure that the valuable insights and advancements in obesity research reach and resonate with the public. In doing so, we could enhance public awareness, encourage informed decision-making, and facilitate meaningful interventions to combat the obesity epidemic in Brazil.

The evidence indicates an increasing scholarly focus on obesity research in Brazil. The increasing trends in both total publications and citations suggest a growing body of research addressing obesity in Brazil and an increasing recognition of this work within the scientific community. The rise in grants and clinical trials further underscores the increasing allocation of resources towards this area of study.

The increase in publications with attention and mean Altmetric Attention Score indicates that the general public is becoming more engaged with scientific research on obesity. However, the decrease in the median Altmetric Attention Score suggests that this increased attention may be driven by a small number of highly popular publications, while the typical publication is receiving less attention over time.

The decrease in median citations indicates that while the total number of citations is increasing, the typical publication is being cited less frequently over time. This could be due to a growing body of literature on obesity, making it harder for individual publications to stand out and garner citations. Over the years, there may have been a saturation of research on obesity-related topics in Brazil. As more studies are conducted and published, it becomes increasingly challenging for individual publications to stand out and receive a high number of citations. Changes in citation behavior among researchers and journals can impact citation counts. Researchers may cite a smaller number of papers due to a focus on recent or highly influential work, which can affect the median number of citations. Additionally, it is possible that the decrease in the median number of citations per publication on obesity in Brazil is due to a specific factor related to the Brazilian research landscape. For example, it is possible that Brazilian researchers are less likely to cite each other's work, or that Brazilian researchers are more likely to publish their work in non-English journals, which are less likely to be cited by researchers from other countries.

The decreasing trends in online image trends, web search trends, and Youtube trends suggest a decline in the general public's interest in obesity. This could be due to a variety of factors such as changes in public perception, competing health interests, or shifts in media coverage. The stability of online news trends suggests that news coverage of obesity has remained consistent over time.

Interestingly, this decline in public interest occurred despite a significant increase in social media usage in Brazil during the study period. According to data from DataReportal – Global Digital Insights, the number of social media users increased from 96 million in 2015 (47 % of the population) to 152.4 million (71.6 % of the population) [23]. This dramatic increase in social media adoption could have potentially influenced online engagement with obesity-related content. However, our findings suggest that increased social media use did not translate into greater public interest in obesity-related topics. This disconnect further emphasizes the need for more effective strategies to engage the public on important health issues like obesity through these increasingly popular platforms.

Recent developments in the pharmaceutical industry, particularly in anti-obesity drugs, may also influence the landscape of obesity research and public awareness in Brazil. For instance, the approval of new weight-loss medications such as semaglutide (Wegovy) in 2021 has garnered significant attention globally [24]. While not yet approved in Brazil as of 2023, discussions around such drugs could potentially impact both scientific research priorities and public interest in obesity-related topics. Future studies should consider how these pharmaceutical advancements might shape the nexus between obesity research and public awareness.

While this study focuses on Brazil, it's worth considering whether similar trends are observed in countries with different socioeconomic backgrounds. Studies in different countries on the relationship between obesity research and public awareness may differ due to varying levels of health literacy, communication gaps, information overload, and public health priorities. For example, a survey on how the public in Germany understood obesity revealed that only a moderate number of people accepted the idea that it was a disease [25]. Future research could benefit from cross-country comparisons to identify global patterns and country-specific factors influencing the translation of obesity research to public awareness.

### 4.2 Factors contributing to the gap in scientific knowledge translation

The observed discrepancy between the increasing scientific interest in obesity and the decreasing public interest in the topic raises important questions about the factors responsible for this gap in the translation of scientific knowledge. Several factors could contribute to this phenomenon.

**Health literacy.** One significant factor could be the level of health literacy among the general population. Health literacy issues, including difficulties in accessing, understanding, and communicating health information, may hinder the public's ability to engage with scientific research effectively [26]. Inadequate health literacy levels have been identified as a challenge, with many individuals relying on sources other than health professionals for information [27]. Efforts have been made to improve health literacy through intervention programs, which have shown some positive impact on health practices and outcomes [28]. Efforts to improve health literacy, particularly in disadvantaged communities, are essential to bridge this gap.

**Communication Gap:** There may be a disconnect in how scientific findings are communicated to the public [29]. Complex scientific language and technical terminology can create barriers to understanding for non-experts. Healthcare professionals and researchers have a crucial role in translating complex scientific knowledge into accessible language for the general audience [30]. Additional community activities and health education initiatives can aid in closing this communication gap [31].

**Information Overload:** The increase in scientific publications over the years might contribute to information overload, making it challenging for individuals to identify and access relevant research findings [32]. Efforts to streamline information dissemination and provide accessible summaries of research can help individuals navigate this wealth of information more effectively. Moreover, educating individuals on how to manage information overload can help them navigate this wealth of information more effectively [33].

**Lack of Public Engagement:** The decrease in public interest may also be attributed to a lack of engaging and interactive platforms for public discussions on obesity-related topics [34]. Creating spaces for informed public discourse and involvement can stimulate interest and awareness.

### 4.3 Solutions to bridge the gap

Addressing the gap in the translation of scientific knowledge to the general population is critical for public health and effective policymaking. Some potential solutions include:

**Health Education Initiatives:** Implementing comprehensive national health education programs that target different age groups and demographics. These programs should focus on improving health literacy and fostering healthy habits. Health professionals can play a central role in delivering these initiatives. Nurses, as trusted healthcare professionals, play a pivotal role in bridging the communication gap between scientific research and the general public. Their direct interactions with patients and communities position them as health educators, communicators, and advocates [35].

**Science Communication:** Encouraging researchers and healthcare professionals to engage in science communication. This involves translating complex research findings into accessible language through public talks, articles, and social media platforms. Collaboration between scientists and science communicators can enhance the reach and impact of research. The study of Brownell et al. suggests that scientists need to receive formal training in science communication to communicate with a diversity of audiences effectively [36]. The study recommends that science communication be included as part of formal scientific training for undergraduate and graduate students.

**Interactive Platforms:** Creating online and offline platforms that facilitate discussions and knowledge-sharing on obesity-related topics. These platforms can include forums, webinars, and community-based events where individuals can engage with experts and each other. The implementation of an online social network in a remotely delivered weight loss intervention that allows participants access to online discussion boards can boost social support and weight reduction outcomes [37]. Online support forums can provide a sense of community, accountability, and motivation for weight loss [38].

**Public-Private Partnerships:** Collaborate with the private sector to promote public health initiatives and combat conflicting interests, such as the promotion of ultra-processed foods. Public-private partnerships can align incentives towards healthier practices [39].

**Multifaceted Approaches:** Recognize that addressing the gap in knowledge translation requires a multifaceted approach. Stigma reduction, empowerment of health teams, development of instructional materials, and awareness campaigns all play a role in bridging this divide. Arts-based approaches, as highlighted in a rapid review by the RAND Corporation, provide innovative and creative ways to engage the public in discussions on research topics [40]. Incorporating art, music, and other creative mediums into health education initiatives can make scientific knowledge more accessible and engaging to a broader audience [41]. These approaches can stimulate interest, awareness, and meaningful conversations surrounding obesity and related health concerns.

## 5 Limitations and potential biases

This study has several limitations that should be considered when interpreting the results. First, the use of Google Trends data may introduce bias towards internet users and may not fully capture offline interest in obesity-related topics. Additionally, changes in internet access and usage patterns over the study period could influence the observed trends. Second, our analysis of scientific publications may be subject to publication bias, where positive or novel findings are more likely to be published. Third, while web searches provide valuable insights into public interest, they may not capture the full scope of public awareness. Other forms of engagement, such as participation in health programs or discussions in non-digital forums, are not reflected in this data. Lastly, the Altmetric Attention Score, while useful for gauging online engagement, has its own limitations and may not fully represent the impact or reach of scientific publications in all contexts.

## 6 Conclusion

In conclusion, the increasing scientific interest in obesity research in Brazil, as evidenced by rising publications and citations, must be complemented by effective knowledge translation to the general population. The gap between scientific knowledge and public awareness can be addressed through improved health literacy, enhanced science communication, interactive platforms, and comprehensive public health initiatives. Closing this gap is essential for informed decision-making, public engagement, and meaningful interventions in the fight against obesity in Brazil.

## Key points

• Obesity in Brazil: A major public health concern demanding attention and action.
• Research-Public Interest Gap: Growing scientific interest, declining public interest.
• Contributing Factors: Socioeconomic, lifestyle, and demographic factors play a role.
• Bridging the Gap: Solutions include education and science communication.

## Data availability

Data included in article/supplementary material is referenced in the article.

## CRediT authorship contribution statement

**Sofia Lafetá Pinto Santos:** Writing – review & editing, Visualization, Validation, Methodology, Investigation, Formal analysis. **Renata Miyabara:** Writing – review & editing, Visualization, Validation, Formal analysis, Data curation. **Rym Ghimouz:** Writing – review & editing, Visualization, Validation, Investigation, Formal analysis, Data curation. **Mirela Dobre:** Writing – review & editing, Visualization, Validation, Supervision, Conceptualization. **Andrei Brateanu:** Writing – review & editing, Visualization, Validation, Supervision, Conceptualization. **Luciana Aparecida Campos:** Writing – original draft, Visualization, Validation, Supervision, Resources, Project administration, Methodology, Conceptualization. **Ovidiu Constantin Baltatu:** Writing – original draft, Visualization, Validation, Supervision, Project administration, Methodology, Conceptualization.

## Declaration of generative AI and AI-assisted technologies in the writing process

During the preparation of this work, the authors used several tools including Microsoft Copilot, QuillBot for scientific writing. After using this tools/services, the authors reviewed and edited the content as needed and take full responsibility for the content of the publication.

## Declaration of competing interest

The authors declare that they have no known competing financial interests or personal relationships that could have appeared to influence the work reported in this paper.
